# The Hair of the Dog

**Published:** 1899-09

**Authors:** 


THE HAIR OF THE DOG.—THE "DRUNK" MICROBE.—"If Dr. L. Maramaldi, of Naples (*Giornale internazionale delle scienze mediche,* 1898, No. 17; *Centralblatt fur innere Medicin,* July 8, 1899), is correct, it is practicable to generate in the blood some substances which will serve as antitoxines to other than the "morbid" poisons. Such a substance he thinks he has formed by using progressively increasing doses of alcohol on the dog. The serum of such a dog he has used successfully in saving other dogs from the effects of doses of alcohol that would otherwise have proved fatal. If his experiments are corroborated, perhaps many an old drunkard may yet be made to serve a good purpose."—*Journal American Medical Association.*

